# The Best Disinfectant

**Published:** 1884-10

**Authors:** John Avery


The Best Disinfectant.—As ordinarily used, disinfectants lull into false security, simply disguising one bad smell by substituting another. To attempt to purify the air of a sick-room made foul by the exhalations of the breath by the use of carbolic acid, etc., is simply to add to the danger instead of lessening it. *Pure air in abundance, circulating freely, is the best disinfectant.* Nothing else can take its place, and it costs nothing, except the effort to open the window. Fresh air is as necessary at night as in day, and is as pure. Additional covering may be required, but no less air.—John Avery, M.D., in *The Sanitarian.*

---

Information Wanted.—Some time ago a homœopathic physician published in an American journal some symptoms produced in a patient by one of Fincke's high potencies of *Symphytum.* Can any one give me the reference?

